# Integrating Deloading into Strength and Physique Sports Training Programmes: An International Delphi Consensus Approach

**DOI:** 10.1186/s40798-023-00633-0

**Published:** 2023-09-21

**Authors:** Lee Bell, Ben William Strafford, Max Coleman, Patroklos Androulakis Korakakis, David Nolan

**Affiliations:** 1 Department of Sport and Physical Activity, Sheffield Hallam University, Sheffield, S10 2BP UK; 2 Department of Exercise Science and Recreation, Applied Muscle Development Laboratory, CUNY Lehman College, Bronx, NY USA; 3 School of Health & Human Performance, Dublin City University, Dublin, Ireland

## Abstract

**Background:**

Deloading is a ubiquitous yet under-researched strategy within strength and physique training. How deloading should be integrated into the training programme to elicit optimal training outcomes is unknown. To aid its potential integration, this study established consensus around design principles for integrating deloading in strength and physique training programmes using expert opinion and practical experience.

**Methods:**

Expert strength and physique coaches were invited to an online Delphi consisting of 3 rounds. Thirty-four coaches completed the first round, 29 completed the second round, and 21 completed the third round of a Delphi questionnaire. In the first round, coaches answered 15 open-ended questions from four categories: 1: General Perceptions of Deloading; 2: Potential Applications of Deloading; 3: Designing and Implementing Deloading; and 4: Creating an Inclusive Deloading Training Environment. First-round responses were analyzed using reflexive thematic analysis, resulting in 138 statements organized into four domains. In the second and third rounds, coaches rated each statement using a four-point Likert scale, and collective agreement or disagreement was calculated.

**Results:**

Stability of consensus was achieved across specific aspects of the four categories. Findings from the final round were used to develop the design principles, which reflect the consensus achieved.

**Conclusions:**

This study develops consensus on design principles for integrating deloading into strength and physique sports training programmes. A consensus definition is proposed: “Deloading is a period of reduced training stress designed to mitigate physiological and psychological fatigue, promote recovery, and enhance preparedness for subsequent training.” These findings contribute novel knowledge that might advance the current understanding of deloading in strength and physique sports.

**Supplementary Information:**

The online version contains supplementary material available at 10.1186/s40798-023-00633-0.

## Background

To achieve optimal athletic performance at select time points relative to the competition schedule, strength (e.g., powerlifting, weightlifting, strongman/woman) and physique (e.g., bodybuilding) athletes will participate in strategically-planned resistance exercise training, organized in a cyclic manner [1, 2]. Such training typically involves undertaking periods of challenging training above the habitual level, designed to invoke a physiological adaptation, and periods of reduced training stress, designed to reduce fatigue and mitigate the risk of maladaptation [3]. Indeed, short-term periods of challenging training (facilitated through an increase in training volume or intensity) followed by a period of reduced training stress can lead to improved performance. However, continuous periods of challenging resistance exercise training without enough recovery can disturb the athlete’s physical and psychological well-being, leading to non-functional overreaching (NFOR) or theoretically the overtraining syndrome (OTS) [4–6]. Periods of reduced training stress involve a standardized decrease in the quantity of training [7] and can occur either within the overall training macrocycle (e.g., during the off-season), between or during training mesocycles (e.g., lower training stress weeks), or within a training microcycle (e.g., easier training sessions) [8].

Strength athletes often participate in tapering; a period of reduced training stress in the days/weeks prior to a competition, designed to optimize specific fitness characteristics, i.e., peaking [9]. Previous research has indicated that as many as 87–99% of competitive strength athletes incorporate a taper into their programme [10, 11]. In sports such as weightlifting and powerlifting, tapering involves a reduction in training volume while maintaining or slightly reducing training intensity [11, 12] and is generally undertaken for a period of ~ 7 days, with the final training session taking place 4 ± 2 days prior to the competition date [12, 13]. Unlike strength sports, physique sports athletes do not normally incorporate a taper into their training programme [14]. Instead, these athletes will maintain normal resistance exercise training while manipulating energy intake, macronutrient composition, hydration levels, and general physical activity levels (e.g., increased cardiovascular exercise) in the days prior to competition to achieve peak aesthetic condition [1, 14]. The general non-use of tapering in physique sports is likely due to the emphasis on aesthetic condition rather than athletic performance [1, 15].

The terms “regeneration microcycles,” “lighter weeks,” “unloading weeks,” “restitution/recovery weeks,'' and “deloading” have all been used to describe phases of reduced training stress that occur across the overall training programme, but not immediately prior to competition [16–21]. The objective of these training phases is to mitigate fatigue, promote recovery, and reduce the risk of NFOR/OTS [16–18]. Unlike the taper, the objective of these phases is not to achieve peak performance, but to enhance preparedness for the *subsequent* training cycle so that the athlete can “reload” and “push again.” [22]. Although the terminology is often used interchangeably by strength and physique coaches, tapering can, therefore, be differentiated from other phases of reduced training stress by both positionality and overall objective [22].

Although no clear consensus definition exists, deloading has been described as a short-term period of reduced training volume and intensity designed to mitigate fatigue and improve training outcomes [21]. To date, there is no research that has objectively reported the prevalence of deloading within strength or physique sports. However, its utilization within strength and physique sports is ubiquitous [22]. Deloading is most likely to be integrated into the athlete’s training programme at the end of each training mesocycle or following an “impact microcycle” e.g., a planned overreaching microcycle [20, 22]. Deloading is generally undertaken every 4–6 weeks for a period of ~ 7 days, although some deloads might range in duration from a singular training session to 2 weeks [22]. During a deloading phase, strength and physique coaches will normally decrease training volume by reducing the number of repetitions completed within a set, the number of sets completed within a training session, or a combination of these strategies [20, 22]. Coaches might also reduce the overall intensity of effort by decreasing the percentage of one-repetition maximum (1-RM) or stopping sets further from muscular failure (i.e., increasing repetitions in reserve). Additionally, exercise selection and configuration might be altered by the coach to reduce training monotony and “change things up” [22]. Overall, empirical research investigating the organization of training variables during deloading is both disparate and heterogeneous, and it is evident that coaches approach the implementation of deloading in a pragmatic and individualized way [22]. Therefore, more research is required to assist both practitioners and sports scientists better understand the factors that influence the design and integration of deloading into strength and physique sport training programmes.

The value of coaches’ experiential knowledge has been neglected in traditional sports science and sport coaching research, resulting in a considerable gap between science and good practice [23]. This might be, in part, due to the complexity of studying athletic populations in situ using classical empirical research designs [24]. However, without guidance from experienced coaches and practitioners, research may not fully elucidate the complicated, multifactorial nature of resistance exercise training prescription. Consequently, improving communication between experimental research and applied environments will foster robust coaching practices, particularly in under-investigated domains such as deloading, where the existing literature is limited [22].

To gain expert consensus on a novel topic within sports science, the Delphi method has previously been utilized [25–29]. This method involves a panel of experts responding anonymously to a series of iterative questionnaires, with feedback from respondents used between rounds to reach a consensus within the group [30, 31]. Given the paucity of research investigating deloading (and the importance of understanding its utility within strength and physique sports), a Delphi method is considered an appropriate methodological tool to enhance knowledge in this domain. Deloading represents a novel area of research, therefore, the aim of this study was to utilize a Delphi method to establish a set of design principles for the integration of deloading into strength and physique sports training programmes.

## Methods

### Study Design

An online-Delphi study utilizing three iterative rounds was undertaken [32]. Each round included an ad-hoc questionnaire which was developed and administered using a commercial survey provider (Qualtrics©, Provo, Utah, United States). To uphold rigour throughout the Delphi process, the authors selected the exclusion and inclusion criteria for sampling ‘experts’, the thresholds for consensus, the number of rounds, and the analytical approach before recruiting participants [33]. In making these decisions, the authors were informed by a pragmatic approach that addressed the research aims centrally, emphasizing the transferability of findings to coaching practice in strength and physique sports environments, and shared meaning and communication in disseminating new knowledge [34].

### Panel Selection

Coaches with expertise in strength and/or physique sports were selected for this study. Purposive sampling was used to recruit participants that were associated with contacts from coaching science and strength and conditioning networks and via social media. To be included in the expert panel, coaches were required to have accreditation/certification from a relevant governing body (e.g., National Strength and Conditioning Association (NSCA), United Kingdom Strength and Conditioning Association (UKSCA)) or a university degree in a related subject area (e.g., Sport and Exercise Science), as well as > 3 years of experience coaching either a strength or physique sport(s). For this study, “strength sports” included weightlifting, powerlifting, and strongman/woman. “Physique sports” comprised all forms of bodybuilding (e.g., Classic, Physique, Figure, Bikini). The choice of sports included in each category was based on previous strength and physique sports research [22].

Unlike experimental studies that use statistical power to determine appropriate sample sizes, the sample size in Delphi studies is dependent on the dynamics of the group in reaching consensus, with 10–18 expert respondents considered optimal for consensus to be achieved [35–37]. Sixty participants were invited to participate, with 34 completing the first round (56.7% response rate), 29 of 34 completing the second round (85.3% response rate) and 21 of 29 completing the third round (72.4% response rate). Table 1 outlines the panel demographics. Ethical approval was granted by the university ethics committee of the lead author [ER45112574] in accordance with the principles of the Declaration of Helsinki [38]. All participants provided informed written consent prior to taking part in the study.

**Table 1:** Participant demographics

	Round 1 (*n* = 34)	Round 2 (*n* = 29)	Round 3 (*n* = 21)
*Descriptives:*			
Age (Years) (Mean ± SD)	33.4 ± 7.4	33.4 ± 6.8	34.8 ± 7.3
Coaching experience (Years) (Mean ± SD)	9.1 ± 6.0	8.9 ± 5.5	9.7 ± 6.1
Experience competing as an athlete (Years) (Mean ± SD)	8.0 ± 5.7	7.8 ± 5.3	8.8 ± 5.7
Duration in current occupation (Years) (Mean ± SD)	6.5 ± 5.0	6.4 ± 4.7	6.8 ± 4.8
*Current role:*			
Coach (e.g., Strength and Conditioning, Physique)	73.5% (25)	69.0% (20)	71.4% (15)
Sport Scientist	5.9% (2)	6.9% (2)	9.5% (2)
Other	20.6% (7)	24.1% (7)	19.0% (4)
*Sports currently working with:* *Some coaches denoted multiple sports			
Bodybuilding	55.9% (19)	58.6% (17)	57.1% (12)
Powerlifting	76.5% (26)	79.3% (23)	81.0% (17)
Strongman/woman	11.8% (4)	10.3% (1)	9.5% (2)
Weightlifting	8.8% (3)	3.4% (1)	4.8% (1)
*Sports currently competing in:* *Some coaches denoted multiple sports			
Bodybuilding (including classic physique, bikini etc.)	29.4% (10)	31.0% (9)	23.8% (5)
None	11.8% (4)	10.3% (3)	14.3% (3)
Powerlifting	61.8% (21)	58.6% (17)	52.4% (11)
Strongman/woman	8.8% (3)	10.3% (3)	9.5% (2)
Weightlifting	8.8% (3)	6.9% (2)	4.8% (1)
*Highest level of athlete currently or previously coached*			
Collegiate	5.9% (2)	6.9% (2)	9.5% (2)
International	64.7% (22)	65.5% (19)	66.7% (14)
Olympic	8.8% (3)	6.9% (2)	9.5% (2)
National	17.6% (6)	17.2% (5)	14.3% (3)
Recreational	2.9% (1)	3.4% (1)	0.0% (0)
*Highest level of competition competed at as an athlete*			
Collegiate	8.8% (3)	10.3% (3)	14.3% (3)
International	35.3% (12)	34.5% (10)	28.6% (6)
National	44.1% (15)	41.4% (12)	42.9% (9)
None	5.9% (2)	6.9% (2)	9.5% (2)
Regional	5.9% (2)	6.9% (2)	4.8% (1)
*Highest academic qualifications:*			
No academic degree	11.8% (4)	10.3% (3)	9.5% (2)
Undergraduate degree	20.6% (7)	17.2% (5)	14.3% (3)
Master’s degree	50.0% (17)	51.7% (15)	52.4% (11)
Doctorate degree	17.6% (6)	20.7% (6)	23.8% (5)
*Professional qualification(s):* *Some coaches denoted multiple qualifications			
Strength and Conditioning Accreditation (e.g., NSCA, UKSCA)	20.6% (7)	17.2% (5)	23.8% (5)
Fitness Industry Coaching Qualification (e.g., ACSM-CPT, NASM-CES)	41.2% (14)	44.8% (13)	47.6% (10)
Sports Coaching Qualification (e.g., National Governing Body Qualifications)	17.6% (6)	17.2% (5)	19.0% (4)
Other (e.g., Nutrition Qualification)	11.8% (4)	10.3% (3)	14.3% (3)
None stated	44.1% (15)	41.4% (12)	28.6% (6)
*Country of employment:*			
Australia	2.9% (1)	3.4% (1)	4.8% (1)
Belgium	2.9% (1)	3.4% (1)	4.8% (1)
Canada	5.9% (2)	6.9% (2)	4.8% (1)
Greece	26.5% (9)	20.7% (6)	9.5% (2)
Ireland	5.9% (2)	6.9% (2)	4.8% (1)
Netherlands	2.9% (1)	3.4% (1)	4.8% (1)
New Zealand	2.9% (1)	3.4% (1)	0.0% (0)
United Kingdom	20.6% (7)	20.7% (6)	23.8% (5)
United States	29.4% (10)	31.0% (9)	42.9% (9)

### Procedure

As considered optimal to reach consensus, the online-Delphi procedure sought to reach consensus after three rounds [39]. Participants were required to complete the questionnaire for the preceding round to progress onto subsequent rounds.

**Fig. 1:**
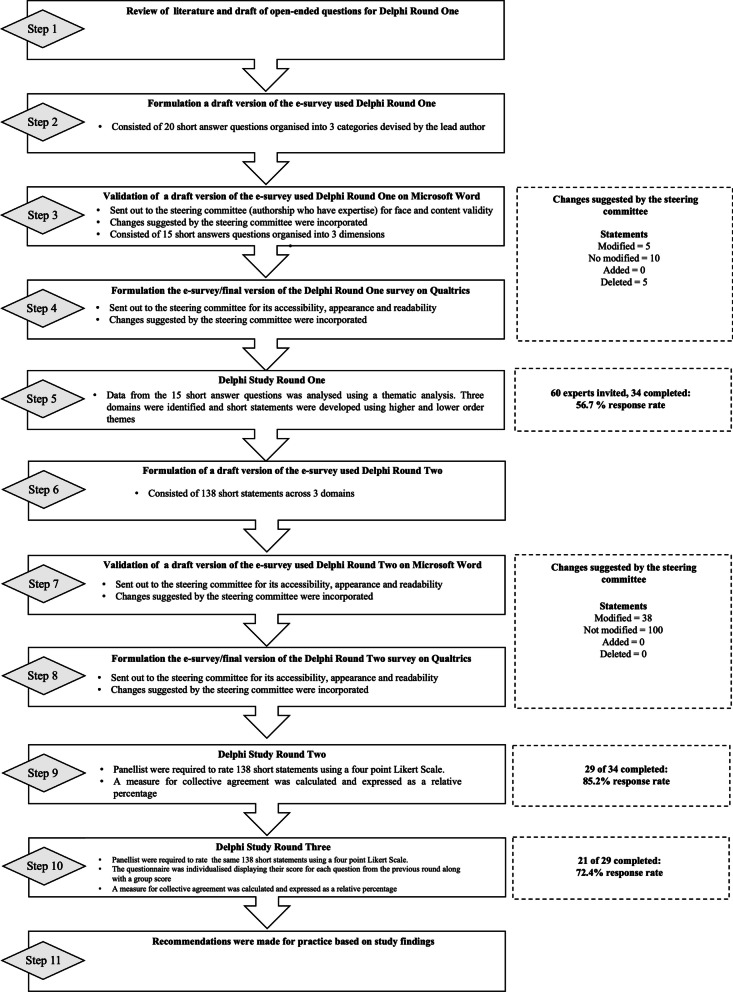
Delphi Procedure. [28] Adapted from Strafford et al.

Round 1: To afford observation of coaches’ experiences and perceptions [40] the first round used open-ended, free-text questions. Fifteen open-ended questions were formulated based on findings from peer-reviewed literature on deloading [22]. The wording of these open-ended questions was informed by the lower-order themes, higher-order themes, and in-text quotations from Bell et al. [22]. Four categories were used to organize the open-ended questions: 1: General Perceptions of Deloading; 2: Potential Applications of Deloading; 3: Designing and Implementing Deloading Training; and 4: Creating an Inclusive Deloading Training Environment. After developing these initial open-ended questions, the lead author met with the other authors to discuss and cross-reference the appropriateness of all questions to the research aim. This afforded dialogue via a collaborative and reflexive working environment where suggestions and ideas from each author listed in the by-line were appraised critically before being integrated into question development where relevant. Questions were either accepted without revisions, developed to omit bias in language or removed from the final question pool. This process enhanced uniformity in question development by ensuring that the language remained as close to the original wording of the concepts and findings outlined in Bell et al. [22] (Fig. 1) [41]. A secure email link, which remained open for four weeks, was used to distribute the online questionnaire for the first round. The list of open-ended questions in the first round is available in the Additional file 1.

Microsoft Excel (Version 19) was used to analyze responses from the first round via a two-stage reflexive thematic analysis [42, 43]. A pragmatic approach to reflexive thematic analysis was selected, including deductive and inductive approaches [42–44]. The first coding stage was a deductive analysis, where free-text responses from the open-ended questions were grouped into four dimensions (General Perceptions of Deloading, Potential Applications of Deloading Training, Designing and Implementing Deloading Training, Creating an Inclusive Deloading Training Environment). The lead author undertook this first coding stage, which included reading the free-text responses multiple times to identify language relating to each dimension. Peer consultation was employed after this first coding stage, where the authorship independently read the responses from the first round and then engaged in open discussion on the initial dimensions determined by the lead author.

By aligning with pragmatism, the authors recognized that knowledge could not be “theory-free” in that knowledge can be both explicit (as with a theoretical understanding of the subject) and implicit (as with knowledge of how to do things from experience) [45]. Therefore, after data were organized into the three dimensions, a second coding stage was undertaken, consisting of deductive and inductive analyses [46]. This reflexive and collaborative approach to the thematic analysis process was used to gain a more nuanced and richer interpretation of the data rather than gain consensus on meaning [43]. Initial codes generated from the reflexive thematic analysis of first-round responses were grouped into higher-order and lower-order themes relating to study aims. Next, codes that could have been classified into multiple themes were grouped into the theme that best fit. In 4 dimensions, 14 higher-order and 138 lower-order themes were highlighted from the reflexive thematic analysis of free-text responses.

Round 2: The lead author used the language from the first round of free-text responses and the higher- and lower-order themes from the reflexive thematic analysis to develop 138 short statements which were organized into four dimensions: (1) General Perceptions of Deloading, (2) Potential Applications of Deloading Training, (3) Designing and Implementing Deloading Training, (4) Creating an Inclusive Deloading Training Environment. Developing these short statements consisted of the lead author writing one idea per statement as an action to ensure minimum overlap with other items and omit ambiguity [47]. The research team met again to discuss the appropriateness of each statement for addressing the research aims. Draft statements were refined where appropriate to ensure that wording remained as faithful as possible to the original wording of the participants’ free-text responses [41]. For uniformity, statements were either accepted as presented by the lead author, modified to remove bias in language, or deleted (Fig. 1). The open-ended questions in the second and third rounds are available in the Additional file 1.

A secure email link, which remained open for two weeks, was used to distribute the online questionnaire for the second round. Participants were instructed to rate each statement using a four-point Likert scale as either: strongly agree, agree, disagree, or strongly disagree [37]. As a pragmatic decision, an additional option of ‘don’t know’ was also included to afford participants the opportunity to accurately report if they did not have an opinion on a specific statement, rather than it being a requirement to give a substantive perspective option [48]. Raw response data were analyzed descriptively using absolute and relative frequencies.

Round 3: For the third and final round, panellists who responded to the second round received personalized online questionnaires via a secure email link that remained open for two weeks. Each questionnaire included the participant’s individual response from the second round, along with a summary of the group responses as a relative frequency. Taking this approach ensured that participants had the opportunity to revise their answers from the second round if they wished to do so [28]. In that, only statements that reached consensus in the third round and final were used establish a set of design principles for the integration of deloading into strength and physique sports training programmes. Raw response data were analyzed descriptively using absolute and relative frequencies.

Criteria for Consensus: A wide range of consensus levels from 50 to 80% have been used in previous Delphi studies [30]. Following formal consultation with the authorship team and through consultation with previous peer-reviewed work, the consensus was defined as ≥ 70% of the panel agreeing/strongly agreeing or disagreeing/strongly disagreeing with a statement in the third round [37]. All ‘don’t know’ responses were excluded from the analysis to ensure that each statement’s reported percentage agreement or disagreement represented the consensus among panellists who believed they held a firm view [49]. Consensus was considered stable if the variance between the second and third round response varied by ≤ 10%, as recommended by Duffield [50].

## Results

Tables 2, 3, 4, 5 provide an overview of the Delphi statements, including those that reached consensus in the second and third rounds. Findings from the third round were used to develop the recommendations presented in this study, which are reflective of the consensus achieved between coaches of the expert panel. Bold text is used in the tables to note denote statements where ≥70% consensus was achieved; Agreement = agree+strongly agree; Disagreement = disagree+strongly disagree.

**Table 2:** Responses to statements in the general perceptions of deloading dimension

General perceptions of deloading	Round 2 (*n* = 29)		Round 3 (*n* = 21)	
	Agreement (%)	Disagreement (%)	Agreement (%)	Disagreement (%)
*Deloading training typology*				
Deloading could be a reduction in overall training demand	100.0	0.0	**100.0**	**0.0**
Deloading could be a reduction in training intensity	96.6	3.4	**100.0**	**0.0**
Deloading could be a reduction in training volume	96.6	3.4	**100.0**	**0.0**
Deloading could be a reduction in training frequency	89.7	10.3	**100.0**	**0.0**
Deloading could be a reduction in proximity to failure	100.0	0.0	**100.0**	**0.0**
Deloading could be a way of mitigating physical fatigue in the training cycle	100.0	0.0	**100.0**	**0.0**
Deloading could be a way of mitigating psychological fatigue in the training cycle	100.0	0.0	**100.0**	**0.0**
Deloading could be a period of recovery	100.0	0.0	**100.0**	**0.0**
Deloading could be a period of adaptation	78.6	21.4	**85.0**	**15.0**
*Taper or Deload?*				
Deloading is a form of tapering	51.9	48.1	55.0	44.0
Deloading is different from tapering, as tapering decreases training volume but maintains training intensity whereas deloading does not	57.1	42.9	50.0	50.0
Tapering occurs before competition whereas deloading can occur anywhere in the training programme	92.9	7.1	**95.2**	**4.8**
Tapering is designed to achieve peaking whereas deloading is designed to promote recovery	88.9	11.1	**89.5**	**10.5**
*Current education on deloading training*				
Deloading is sufficiently represented in strength and conditioning textbooks	8.0	92.0	**11.1**	**88.9**
Deloading is sufficiently represented in strength and conditioning qualifications	13.6	86.4	**0.0**	**100**
Current approaches to deloading are primarily based on coach experiential knowledge of deloading	96.3	3.7	**100**	**0.0**
There are limited scientific studies and understanding about deloading	100.0	0.0	**100.0**	**0.0**
Scientific studies on deloading would inform my approach to deloading	96.6	3.4	**100.0**	**0.0**
Scientific studies on deloading should be open access	100.0	0.0	**100.0**	**0.0**
Networking would provide opportunities for coaches to learn more about deloading	96.3	3.7	**100.0**	**0.0**
Non-traditional media, like blogs, podcasts and YouTube videos could be used to disseminate knowledge of deloading	100.0	0.0	**100.0**	**0.0**

**Table 3:** Responses to statements in the potential applications of deload training dimension

Potential applications of deload training	Round 2 (*n* = 29)		Round 3 (n*n*= 21)	
	Agreement (%)	Disagreement (%)	Agreement (%)	Disagreement (%)
*Potential reasons to deload an athlete*				
Deloading could increase adherence to normal training	96.4%	3.6%	**100.0%**	**0.0%**
Deloading around competition could allow athletes performance to peak	88.5%	11.5%	**85.0%**	**15.0%**
Deloading could be used to reduce injuries	100.0%	0.0%	**100.0%**	**0.0%**
Deloading could be used to reduce the risk of overreaching	85.7%	14.3%	**95.2%**	**4.8%**
Deloading could be used to reduce the risk of overtraining	92.9%	7.1%	**95.0%**	**5.0%**
Deloading could be used to reduce monotony of training	86.4%	13.6%	**94.7%**	**5.3%**

**Table 4:** Responses to statements in the designing and implementing deload training dimension

Designing and Implementing deload training	Round 2 (*n* = 29)		Round 3 (*n* = 21)	
	Agreement (%)	Disagreement (%)	Agreement (%)	Disagreement (%)
*Approaches to integrating deloading into an athlete’s training*				
Deloading could be coach driven	100.0	0.0	**100.0**	**0.0**
Deloading could be athlete driven	100.0	0.0	**100.0**	**0.0**
Deloading could be data driven	100.0	0.0	**100.0**	**0.0**
Deloading could be a natural break in training	96.6	3.4	**100.0**	**0.0**
Deloading could be used so intensity of training can be increased afterwards	92.9	7.1	**100.0**	**0.0**
Deloading could be used so new training blocks and exercises can be introduced	96.4	3.6	**100.0**	**0.0**
Deloading could occur in the first week of a new mesocycle	67.9	32.1	**75.0**	**25.0**
Deloading could occur in the last week of a mesocycle	96.6	3.4	**95.2**	**4.8**
Deloading could occur at the beginning, middle and end of the mesocycle	69.0	31.0	**76.2**	**23.8**
Deloading could be introduced between mesocycles of training	96.4	3.6	**100.0**	**0.0**
The timing of deloading depends on competition	93.1	6.9	**95.2**	**4.8**
Deloading could be planned into the normal training cycle when preparing athletes for competition	100.0	0.0	**100.0**	**0.0**
Deloading could only be used if training throughout the entire macrocycle is sufficient to drive adaptations	42.3	57.7	50.0	50.0
Deloading could occur when athletes feel physically and mentally fatigued regardless of training week in the mesocycle	89.7	10.3	**100.0**	**0.0**
All parts of the training programme could be deloads	37.0	63.0	33.3	66.7
Deloading could be difficult to implement due to sport demands	67.9	32.1	**75.0**	**25.0**
*Deload training volume*				
Deloading could be used to maintain fitness	75.0	25.0	**89.5**	**10.5**
Deloading could reduce training volume by cutting days training	93.1	6.9	**100.0**	**0.0**
Deloading could reduce volume by lowering sets	100.0	0.0	**100.0**	**0.0**
Deloading could reduce volume by lowering reps	96.6	3.4	**100.0**	**0.0**
Deloading could reduce volume from maintenance level	77.8	22.2	**95.0**	**5.0**
Deloading could use a minimum effective dose for volume	93.1	6.9	**90.5**	**9.5**
*Deload training intensity of effort*				
Training intensity could remain high during the deload	69.0	31.0	**81.0**	**19.0**
Training intensity during the deload may not be the same as normal training	89.7	10.3	**100.0**	**0.0**
Deloading could reduce sets to lower training intensity	81.5	18.5	**85.0**	**15.0**
Deloading could reduce reps to lower training intensity	93.1	6.9	**95.2**	**4.8**
Fatigue is related to training intensity	78.6	21.4	**81.0**	**19.0**
Training intensity during the deload may depend on the demand of the sport	100.0	0.0	**100.0**	**0.0**
Training intensity during the deload may depend on if the athlete is male or female	64.0	36.0	66.7	33.3
Training intensity during the deload may depend on the age of the athlete	77.8	22.2	**80.0**	**20.0**
Training intensity during the deload may be set using basic exercises	83.3	16.7	**94.4**	**5.6**
Training intensity could remain the same during the deload, but the volume may drop	86.2	13.8	**95.2**	**4.8**
Deload training intensity could be lower during the first week of a new mesocycle	84.0	16.0	**84.2**	**15.8**
Deload training intensity could drop over consecutive days	92.3	7.7	**100.0**	**0.0**
Deload training intensity could remain the same as during normal training	69.0	31.0	**76.2**	**23.8**
During competitive periods volume and training intensity during the deload may be high to allow recovery	10.7	89.3	**19.0**	**81.0**
During non-competitive periods volume and training intensity during the deload could decrease	93.1	6.9	**100.0**	**0.0**
*Deload training exercise selection*				
During deloading exercise selection could remain unchanged	96.6	3.4	**100.0**	**0.0**
During deloading the exercise selection may be the athlete decision	75.9	24.1	**81.0**	**19.0**
During deloading new exercises could be introduced	79.3	20.7	**81.0**	**19.0**
During deloading, exercise complexity could be introduced	37.0	63.0	45.0	55.0
During deloading, ‘pivot blocks’ could be introduced	81.8	18.2	**86.7**	**13.3**
During deloading, main exercises could be trained with lower volume or training intensity	100.0	0.0	**100.0**	**0.0**
Deloading could use the same exercises to avoid muscle soreness caused by novel stimulus	100.0	0.0	**100.0**	**0.0**
Deloading could introduce new exercises that will be used in the next mesocycle	89.7	10.3	**95.2**	**4.8**
Assistance exercises could be removed during deloading	86.2	13.8	**85.7**	**14.3**
Training accessories could be removed during deloading	82.8	17.2	**85.7**	**14.3**
Exercise selection during deloading could match the upcoming mesocycle	89.7	10.3	**95.2**	**4.8**
Deloading could be used (re)establish technique	89.7	10.3	**95.2**	**4.8**
Deloading could focus on removing/reducing secondary exercises	82.8	17.2	**95.2**	**4.8**
Deloading could integrate accessory movements	77.8	22.2	**85.0**	**15.0**
Deloading reduce volume by decreasing the number of exercises in a training session	92.9	7.1	**95.0**	**5.0**
Deloading reduces volume by omitting power exercises	31.8	68.2	**14.3**	**85.7**
Deloading could maintain training demand	17.9	82.1	**9.5**	**90.5**
Deloading could use familiar exercises	100.0	0.0	**100.0**	**0.0**
Deloading could use new exercises	82.1	17.9	**90.5**	**9.5**
Deloading could focus on technique of main lifts	89.7	10.3	**95.2**	**4.8**
Deloading could keep multijoint exercises	100.0	0.0	**100.0**	**0.0**
Deloading could include activities outside of the gym	85.7	14.3	**100.0**	**0.0**
*Frequency of deloading training*				
Deloading could be included in each mesocycle	96.4	3.6	**100.0**	**0.0**
There may be multiple deloads depending on the mesocycle length	96.4	3.6	**100.0**	**0.0**
Deloading frequency may depend on other sports training	96.6	3.4	**100.0**	**0.0**
If the sport has predominately technical training a deload may not be needed	56.0	44.0	50.0	50.0
Deload frequency may depend on how athlete responses to training stimulus	100.0	0.0	**100.0**	**0.0**
Deloading may occur before, during or at the end of a competition period	96.4	3.6	**95.2**	**4.8**
*Autoregulatory and pre-planned deload training*				
Deloading could be pre-planned	100.0	0.0	**100.0**	**0.0**
Deloading could be pre-planned around lifestyle		96.6	3.4	**100.0**
Deloading could be autoregulatory	100.0	0.0	**100.0**	**0.0**
Deloading could be both pre-planned and autoregulatory	100.0	0.0	**100.0**	**0.0**
The coach may schedule periods where deloads could be taken	100.0	0.0	**100.0**	**0.0**
The athlete may schedule periods where deloads could be taken	86.2	13.8	**95.2**	**4.8**
*Adapting normal training frequency during deload training*				
During deloading normal training frequency may not change unless the athlete’s lifestyle is very busy	85.7	14.3	90.5	9.5
During deloading training frequency may not alter	82.1	17.9	**100.0**	**0.0**
Deloading could reduce the mesocycle duration	72.0	28.0	**77.8**	**22.2**
*Adapting session duration during deload training*				
During deloading, session duration could increase	27.6	72.4	33.3	66.7
During deloading, session duration could decrease		96.6	3.4	**100.0**
During deloading, session duration could remain the same	79.3	20.7	**90.5**	**9.5**
During deloading, sessions may be shorter due to lower volume and intensity	100.0	0.0	**100.0**	**0.0**
During deloading, training volume may be naturally reduced	100.0	0.0	**100.0**	**0.0**
*Deloading as a coach and athlete*				
Deloading may be pre-planned for athletes/clients but more autoregulatory in my own training	69.0	31.0	66.7	33.3
Deloading may be more autoregulatory for athletes/clients but pre-planned but in my own training	50.0	50.0	50.0	50.0
Deloading may be the same for athletes/clients as my own training	89.7	10.3	**100.0**	**0.0**
Deloading could be more cautious when prescribing to athletes compared to my own training	65.5	34.5	**71.4**	**28.6**
For deloading, volume and intensity may change depending on experience, age, and level of performance	100.0	0.0	**100.0**	**0.0**

**Table 5:** Responses to statements in the creating an inclusive deloading training environment dimension

Creating an inclusive deloading training environment	Round 2 (*n* = 29)		Round 3 (*n* = 21)	
	Agreement (%)	Disagreement (%)	Agreement (%)	Disagreement (%)
*Creating an inclusive deloading training environment*				
Deloading may be easier to implement when the sport has infrequent competitions.	100.0	0.0	**100.0**	**0.0**
Deloading may be easier to implement when it can be integrated around sport competitions	85.7	14.3	**90.5**	**9.5**
Deloading may be harder to implement when the sport has frequent competitions	96.4	3.6	**100.0**	**0.0**
Deloading may be harder to implement as athletes find it boring	62.1	37.9	66.7	33.3
Deloads may be hard to implement as athletes love to train	69.0	31.0	**76.2**	**23.8**
Deloading could allow coaches to identify the needs analysis of new athletes	92.3	7.7	**100.0**	**0.0**
Deloading may be harder to implement due to athlete perspectives on what a deload is, versus what it actually includes	88.0	12.0	**100.0**	**0.0**
Deloading may be harder to implement due to the coach’s perspectives on what a deload is, versus what it actually includes	84.0	16.0	**100.0**	**0.0**
Members of a coaching team not working in collaborative way could be a barrier to integrating deloading	96.2	3.8	**100.0**	**0.0**
Lack of athlete education and understanding on a deloading could be barrier to integration	92.9	7.1	**95.0**	**5.0**
Lack of coach education and understanding on a deloading could barrier to integration	100.0	0.0	**100.0**	**0.0**
Age may be a barrier to deloading integration	65.2	34.8	61.1	38.9
Training culture may be a barrier to deloading integration	96.6	3.4	**100.0**	**0.0**
Illness could be a barrier to deloading integration	65.4	34.6	58.8	41.2
Athlete lifestyle could be a barrier to deloading integration	79.3	20.7	**81.0**	**19.0**
Deloading should be different for males and females	20.0	80.0	**15.4**	**84.6**
Developing coach education could resolve barriers for integrating deloading	100.0	0.0	**100.0**	**0.0**
Developing athlete education could resolve barriers for integrating deloading	100.0	0.0	**100.0**	**0.0**
Working a multi-disciplinary team could reduce barriers for integrating deloading	96.0	4.0	**94.7**	**5.3**
Involving athletes in decision making on deloading could aid its integration	100.0	0.0	**100.0**	**0.0**
Research informed practice could aid the integration of deloading	96.6	3.4	**95.2**	**4.8**
Communication between parties may be key to integrating deloading	100.0	0.0	**100.0**	**0.0**
Athlete autonomy may be key for integrating deloading	76.9	23.1	**90.0**	**10.0**
Coaches could focus on relaying the concepts and foundations of deloading to athletes	93.1	6.9	**100.0**	**0.0**
Coaches could focus on encouraging deloading as a training tool rather than a ‘fad’	96.4	3.6	**100.0**	**0.0**
Deloading could be integrated in moderation based on the athlete/client training goal	96.6	3.6	**100.0**	**0.0**
Using consistent terminology when disseminating information on deloading could aid its integration	96.4	3.6	**100.0**	**0.0**

### General Perceptions of Deloading

In this dimension (Table 2), the expert panel considered deloading to be a reduction in overall training demand facilitated through a decrease in either training volume or intensity of effort. It was agreed that deloading might mitigate the risk of both physical and psychological fatigue, while facilitating recovery and adaptation.

According to the panel, deloading could occur anywhere in the training programme, while the taper would only occur only before a competition. There was consensus that while tapering is designed to achieve peak performance, the purpose of deloading is to promote recovery and preparedness. It was viewed that deloading is not sufficiently represented in strength and conditioning textbooks or qualifications. Moreover, deloading is underrepresented in the available scientific literature, with the current approaches to deloading based primarily on coach experiential knowledge. There was consensus that more scientific research should be conducted to enhance current understanding about deloading, and that research should be made open access to the coaching community. Additionally, it was proposed that networking events, blogs, podcasts and social media-based educational platforms would assist in the dissemination of deloading knowledge.

### Potential Applications of Deload Training

In this dimension (Table 3), the panel reached a consensus on the potential benefits of deloading, as well as the methods in which deloading could be integrated into the training programme. It was agreed that deloading could increase adherence to the overall training programme, and reduce the risk of NFOR, OTS, training monotony, and injury, while assisting the athlete in achieving performance peaking.

### Designing and Implementing Deload Training

In this dimension (Table 4), the panel agreed that the integration of deloading should be, in part, led by the coach, the athlete, and the available data. There was consensus that the deloading afforded an opportunity to increase training demand in the subsequent training cycle. It was agreed that deloading could be positioned either in the first, middle or final week of a training mesocycle, but that timing of the deload was dependent upon the competition schedule and sporting demands. Deloading could be planned into the athlete’s normal training cycle or utilized when athletes felt physically or mentally fatigued, regardless of where they were within their current mesocycle.

The panel agreed that training volume during deloading could be reduced (relative to the volume utilized in the previous phase of training). This would be achieved through a decrease in either the number of sets per training session, the number of repetitions per set, or through a reduction in training frequency. There was also agreement that a minimum effective dose could be used and that the duration of each training session during deloading could decrease or remain the same. It was agreed that a decrease in session duration would, in part, be the result of a reduction in training volume and intensity.

Panellists agreed that training intensity could either increase or decrease during deloading. There was consensus that, when reduced, training intensity might be lower during the first week of a new mesocycle or could be decreased over consecutive days. Training intensity could also remain the same during the deload, but only when training volume is reduced. It was also agreed that volume and training intensity might decrease during non-competitive periods.

The experts agreed that training volume and intensity were governed, in part, by the demands of the sport, as well as the level of performance or experience, as well as the age of the athlete. There was consensus that the approach to designing and implementing deloading should not be different for male and female athletes.

In relation to exercise selection, exercises might remain unchanged during deloading, or new exercises could be introduced. The rationale for maintaining exercise selection was to reduce the risk of muscle soreness caused by a novel stimulus. There was agreement that main exercises could be adapted by using a lower volume or lower training intensity and that assistance/accessory exercises could be adapted or removed altogether. During the deload, exercise selection should focus on technique for the main exercise(s), the use of multijoint exercises should be maintained and deloading may include activities outside of the gym environment.

It was agreed that deloading could be integrated into the training programme before, during, or at the end of each mesocycle. The consensus was that there could be multiple deloads depending on the length of the mesocycle. How frequent deloading might be integrated would be, in part, dependent on how the athlete responds to the training stimulus presented to them, as well as on other sport training commitments the athlete may have.

Deloading could be pre-planned and organized around the athlete’s lifestyle or integrated using an autoregulatory approach. It was agreed that both the athlete and coach might be involved in scheduling when deloads occurred within the programme.. However, the consensus was that the coach might be more cautious when prescribing deloading to the athlete compared to their own training.

### Creating an Inclusive Deloading Training Environment

In this dimension (Table 5), the expert panel agreed that deloading might be easier to implement when the sport has infrequent competitions, and easier to implement when deloading can be integrated around the competitive schedule.

Panellists agreed that barriers to the implementation of deloading could exist. For example, deloading may be difficult to implement due to the athlete’s or coach’s perspective on what a deload is, versus what it actually includes. A lack of athlete or coach education and understanding of deloading could also be a barrier to its integration. Additional barriers that reached consensus were if athletes are highly motivated and “love to train,” the athlete's lifestyle, training culture, or members of the coaching team not working in a collaborative way. To overcome these barriers, it was agreed that coach and athlete education, working in a multi-disciplinary team, and involving athletes in the decision-making process (through active communication) might be beneficial. Moreover, the panel concurred that research-informed practice, athlete autonomy, and the use of consistent terminology might also be advantageous.

## Discussion

This study sampled expert opinions from coaches on the integration of deloading into strength and physique sport training programmes. The study systematically gained consensus on factors relating to (1) General Perceptions of Deloading; (2) Potential Applications of Deloading; (3) Designing and Implementing Deloading; and (4), Creating an Inclusive Deloading Training Environment. These findings contribute novel knowledge that will advance the current understanding of deloading in strength and physique sports. Moreover, the results of this study provide the co-creation of new knowledge and understanding between coaches and sports scientists; an important step forward to developing a better understanding of deloading in practice. The results of this study will guide coaches in the integration of deloading in a practical environment and researchers in the development of controlled deloading programmes for scientific research purposes. Additionally, this study also highlights the need for future empirical research in this domain.

### The Applications and Objectives of Deloading

A key point of consensus amongst coaches was related to the overall objectives of deloading. Coaches agreed that the purpose of deloading is not to enhance performance per se but to mitigate physiological and psychological fatigue, promote recovery and facilitate physiological adaptation. Moreover, coaches agreed that deloading aims to enhance preparedness for the subsequent training cycle. This rationale is in concordance with the existing (albeit limited) literature [16–18, 20–22]. Panellists of this study agreed that tapering occurs before a competition, but deloading can occur anywhere in the training programme (i.e., the first, middle, or final week of a training mesocycle). Similar to deloading, the taper is designed to reduce training-induced fatigue while retaining training adaptations [7, 51, 52]. However, the taper only occurs in the final period of training before a major competition and is of paramount importance to an athlete’s competition performance [9, 51]. Consequently, while deloading and tapering share similar structural similarities (i.e., the manipulation of training variables to reduce training-related stress), deloading and tapering are different aspects of training.

Applying consistent terminology to deloading was a key point of agreement between panellists. Currently, no clear definition exists but is critical to propel research in the field. This, in part, might explain the misinterpretation of what is (and is not) deloading, and the often interchangeable use of the terms deloading and tapering [22]. Not only would the development of an operational definition allow for clear differentiation between tapering and deloading, but it would also provide a model by which deloading can be researched for the purpose of scientific inquiry. Therefore, based on expert opinion generated from this study, information synthesized from previous research exploring coaches’ perceptions of deloading, information obtained from relevant previous literature [21, 22] and the authors’ own interpretation, we propose the following definition:

Deloading is a period of reduced training stress designed to mitigate physiological and psychological fatigue, promote recovery, and enhance preparedness for subsequent training.

Coaches agreed that deloading reduces the risk of NFOR, OTS, training monotony and training-related injury. Previous research has elucidated that while short-term periods of highly-demanding resistance exercise training can lead to improvements in both muscular strength and hypertrophy (relative to baseline), chronic periods of training can lead to NFOR and theoretically, the OTS [4, 5]. Indeed, risk factors for the development of NFOR and OTS include undertaking prolonged periods of high-volume and/or high-intensity resistance exercise training without sufficient fuelling or recovery, frequent training to muscular failure/high repeated efforts, and participating in monotonous training with limited variation in training demand or exercise selection [4–6, 53]. However, the incidence of NFOR in strength sports and resistance-trained populations is low, even following deliberate attempts to induce maladaptation [4, 5]. Moreover, coaches are not typically concerned that excessive training will result in long-term performance impairment [54]. It is currently unclear whether deloading is a necessary part of the training programme to avoid the deleterious effects of NFOR/OTS.

It has been speculated that prolonged periods of training without sufficient recovery may also contribute to the risk of training-related injury [55]. Further, separating blocks of demanding training with periods of deloading might mitigate the risk of acute joint or musculotendinous injury and subclinical tissue damage [56, 57]. Indeed, it is long-term exposure to training that is assumed to influence injury occurrence, not isolated or single training bouts [55, 58]. However, the relationship between training, performance and injury is complex and multifaceted [59]. Research investigating the impact of training load on the relative risk of injury in strength and physique sports is limited, and most of the available studies in this domain are of low methodological quality [60]. Therefore, while it is plausible to consider that deloading might provide prophylactic benefits to the strength and physique athlete, more research on the epidemiology of injury and changes in risk due to continuous exposure to training needs to be undertaken [61].

### Integrating Deloading into the Strength and Physique Athlete’s Training Programme

The panel of coaches agreed that deloading could be integrated into the training programme through alterations in training volume, training intensity or exercise selection. This multifaceted approach to the design of deloading has also been observed in the available literature, where deloads have been implemented through a decrease in repetitions per set or sets per training session [20, 21, 62–65], a reduction in absolute or relative training intensity [15, 62, 66], or through alterations in exercise selection and configuration [15, 64]. Overall, the variable approach to deloading reported by the expert panel of this research suggests that there is no standardized way to design and integrate deloading into the strength and physique athlete’s training programme. However, there was universal agreement that training volume should be decreased during deloading. Previous literature has shown that undertaking extended periods of high-volume resistance exercise training can result in NFOR [4] and that integrating short-term periods of low training volume can still be effective in maintaining or promoting meaningful increases in muscular strength and hypertrophy, even in resistance-trained individuals and competitive strength athletes [67, 68]. This is perhaps why, in part, coaches involved in this study agreed that a minimum effective dose for volume might be adopted during deloading.

Coaches agreed that while deloading might be positioned in the final week of the mesocycle, it can also be placed in the first week of a new mesocycle. Typically, a new mesocycle of training will incorporate new exercises [8] emphasizing skill and technique development and approaching training in a way that focuses on quality rather than quantity [69]. Previous research [22] has highlighted that deloading presents an opportunity for the athlete to develop new techniques and the incorporation of novel exercises, therefore, there is a rationale for the deloading to occur in the first week of a new mesocycle. Consequently, deloading might incorporate some degree of novelty as a method to reduce training monotony and develop new skills in preparation for the subsequent mesocycle. However, caution should be taken when adjusting exercise selection due to a potential increase in muscle soreness caused by unaccustomed resistance exercise training [70]. Therefore, incorporating new exercises into the deload, with concomitant reductions in training volumes and/or relative intensities, could allow for the integration of novel exercise selection while mitigating the risk of training-related muscle soreness.

Training programmes aiming to enhance muscular strength or hypertrophy have traditionally adopted a periodized approach, where training is organized into a series of training cycles separated by phases of reduced training stress [2, 16, 18, 71]. Strength and physique training programmes are traditionally modeled on a predicted pattern of response to training stress, i.e., the stimulus-fatigue-recovery-adaptation model [17, 72]. Indeed, it is common within strength and physique sports training programmes to adopt a pre-planned approach whereby training is gradually progressed each week of the mesocycle until a deload is applied in the final week [20, 64]. In this sense, regular (every 4–8 weeks) pre-planned deloading serves a precautionary purpose and is likely based on the assumption that phases of reduced training stress are required to allow physiological adaptation to occur [3, 17]. However, while there is evidence to suggest that systematic variation of training can lead to improvements in select measures of athletic performance [16, 18], there is limited evidence to suggest that pre-planned, periodized training is superior to non-periodized training [72–74]. Consequently, regular pre-planned periods of reduced training stress might not be necessary, and this is perhaps the reason why some strength and physique coaches choose not to pre-plan a deload or do not consider it necessary to prescribe them rigidly [22]. It is also worth noting that periodization of resistance exercise training volume and intensity does not seemingly lead to greater muscular hypertrophy compared to non-periodized training [75, 76]. Moreover, none of the studies included in reviews exploring the effects of periodized training on muscular hypertrophy have been designed to directly enhance hypertrophy as the principal outcome [75, 76]. Only strength training protocols have been meta-analyzed for their impact on hypertrophy. Therefore, it is currently unclear whether competitive physique athletes intending to develop muscular hypertrophy for aesthetic reasons should periodize their training [77].

There are very few empirical studies that have investigated the effects of continuous training (training without deloading) versus periodic training (training blocks that are separated by deloading) [78, 79]. In studies by Ogasawara et al. [78, 79], no statistically significant differences were observed in measures of muscular strength or hypertrophy between a continuous training group and a group integrating a three-week period of training cessation after six weeks of training over either a 15 or 24-week period. Additionally, previous research has speculated that prolonged training without sufficient recovery might lead to a blunting of the anabolic signalling process that underpins the adaptive response to resistance exercise training, and as such, integrating short-term periods of deloading might “resensitize” the hypertrophic response to training [80]. However, the influence of deloading on a possible desensitization/resensitization effect have not been studied and it is currently unknown if deloading enhances the adaptive response to training.

Autoregulation is an emergent method used within resistance exercise training prescription to adjust the training volume and intensity of each session based on individual daily fluctuations in fitness, fatigue, and preparedness [81, 82]. An autoregulated approach avoids adopting pre-planned phases of training, and instead, favours continuous modification of training in response to the athlete’s individual rate of adaptation [83]. In resistance exercise training programmes that emphasize muscular strength or hypertrophy, autoregulation can be applied through alterations in either objective or subjective within-session measures (e.g., ratings of perceived exertion, reps in reserve, velocity-based training) or between-session measures (e.g., countermovement jump, 1-RM) [83, 84]. Therefore, adopting an autoregulated approach allows the strength and physique coach to prescribe heavier or lighter training in an undulating manner, rather than in a rigid or pre-planned way [85]. Previous studies have demonstrated that autoregulation of training variables can lead to improvements in both muscular strength and hypertrophy while also deterring maladaptation [81, 86]. While evidence suggests that utilizing an autoregulated deloading might negate (or at least reduce the necessity) of pre-planned deloads, further research is required to accurately assess its effects on muscular strength and hypertrophy in the subsequent training phase compared to a pre-planned paradigm. Panellists agreed that the integration of deloading into the training programme should be, in part, led by the coach, the athlete, and the individual athlete’s performance data. This is perhaps reflected in the agreement to use an autoregulation approach to deloading as it allows a flexible approach to training where the coach and athletes select the type or difficulty of the training session based on perceived capability to perform (i.e., high fatigue levels or high readiness to train) [82]. In this sense, the coach and athlete can use the available data to triangulate the day’s training.

Overall, deloading should be approached in an individualized, athlete-centred manner, combining practice-based guidelines with experience and tacit knowledge. The athlete’s level of competition, training history, chronological and training age, the importance of competition, and lifestyle factors (e.g., work or family commitments), as well as the demands of the sport and competition schedule, should all be considered when developing deloading training [22]. Coaches should adopt a research-informed approach when integrating deloading into the training programme. Therefore, the strength and physique coach must undertake a thorough needs analysis of the athlete and their sport prior to integrating deloading, using an appropriate framework of practice to properly address the factors that influence the response to training [87].

**Fig. 2:**
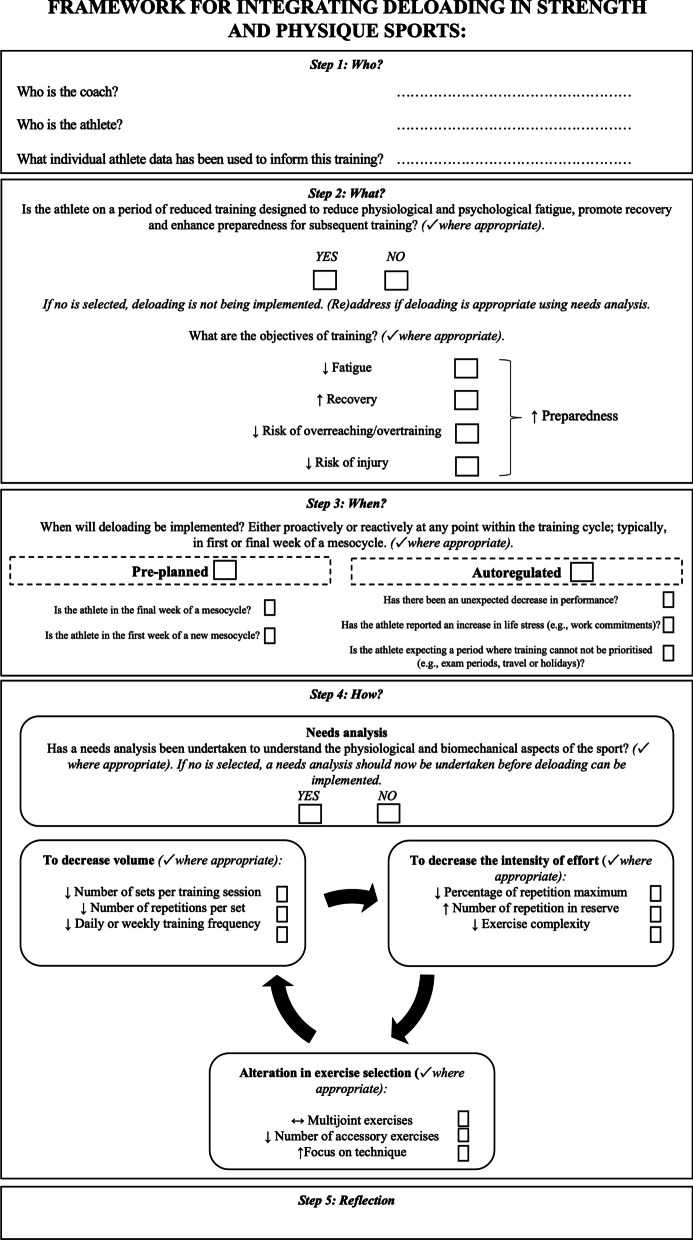
Principles framework for integrating deloading into strength and physique training programmes

Figure 2 provides a resource designed to assist coaches in the integration of deloading into strength and physique training programmes. This resource was developed using statements that reached consensus in Round 3 and was reviewed by all authors to ensure the accuracy of information but also to remain as faithful as possible to the original wording of statements reaching consensus. It is recommended that before integrating deloading into strength and physique training programmes, coaches engage with this resource and relevant coach education material.

### Developing a Collaborative Understanding of Deloading

The value of experiential knowledge for informing strength and conditioning practice has been largely neglected due to difficulties in acquiring data via classic experimental designs. As a result, the rationale for evidence-based knowledge in strength and conditioning has been skewed in the way of limiting the categorization of knowledge to influence practice into ‘what’ in the absence of ‘why’ and ‘how’ [24, 88]. The panel of coaches in this study agreed that deloading research is underrepresented in both published peer-reviewed literature and professional resources (i.e., strength and conditioning textbooks and qualifications). Further, that non-traditional media, such as blogs, podcasts and YouTube videos could be used to disseminate knowledge of deloading.

Advances in theoretical sports science knowledge, and rapid changes in technology to support athlete development, means there is a need for strength and physique coaches to stay up to date with emerging knowledge [89]. Coaches frequently learn from informal sources such as conferences and podcasts as they tend to be contextually relevant, accessible, and applicable to the practical environment [90, 91]. Indeed, while peer-reviewed, published sports science research is used to inform and update practice, coaches are less likely to gain new information directly from scientific sources due to a lack of access (something agreed upon by the expert panel) and a lack of time [90]. This might be, in part, why coaches involved in this research consider current approaches to deloading to be primarily based on coach experiential knowledge of deloading.

Research from Shaw and McNamara [92] has demonstrated that open-access podcasts provide an alternative source of information for coaches and sports scientists due to their convenience, accessibility, and authenticity. As a novel medium of knowledge transfer, educational podcasts provide profession-specific knowledge to increase understanding relating to a specific topic [93]. Moreover, podcast creators often use scientific literature to research for a specific podcast episode [94], therefore the importance of peer-reviewed research cannot be dismissed. However, while podcasts are the preferred vehicle for knowledge transfer in the strength and physique coaching community, it is difficult to verify the legitimacy and accuracy of the information distributed in some podcasts [94].

**Fig. 3:**
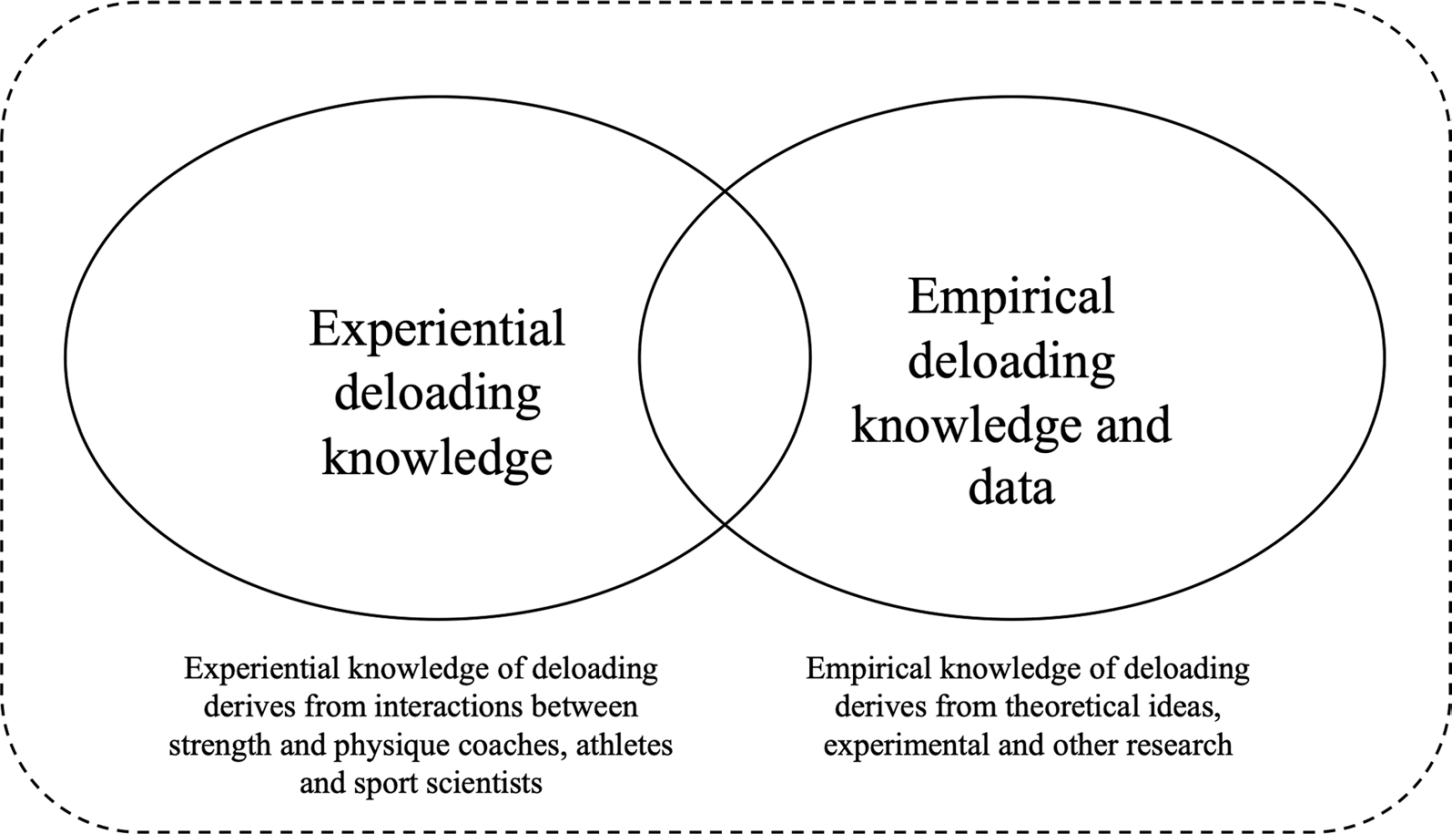
A theoretical framework for enhancing deloading knowledge using a department of methodology approach. (Adapted from Rothwell et al. [24])

Coaches agreed that the current understanding of deloading is governed primarily by experiential knowledge. Given that the coach–sports scientist relationship can contribute to establishing optimal practices in high-performance sporting environments and enhance the transfer of knowledge [95], coaches’ knowledge should serve as a starting point for the development of deloading protocols used for the design and dissemination of scientific findings. The integration of coach experiential knowledge (i.e., knowledge gained by ‘doing’ [96]), and empirical knowledge on deloading is displayed in Fig. 3, with the overlap of the two bodies of knowledge being the result of collaboration between coaches and sports scientists in a ‘department of methodology’ [24]. Over time, it is anticipated that this overlap may lead to the enhancement of experimental research within applied environments and assist in the development of robust deloading practices through collaborative design, shared principles and unified language [24].

While this study assists in developing an understanding of the integration of deloading into strength and physique sports, we acknowledge some potential limitations of this study. The Delphi approach has previously been criticized for its potential for issues in achieving expert selection, researcher bias, and restrictive communication methods [97]. Adopting a pragmatic approach to the Delphi process, the authors acknowledged potential limitations during decision-making, with the best attempts made to uphold rigour in the planning and delivery of the Delphi study. The authors consulted peer-reviewed research when making decisions, for example when deciding the inclusion and exclusion of selected ‘experts', the number of rounds, the analytical approach and thresholds for consensus before the study commenced [33]. Aligned with a pragmatic approach, a ‘don't know’ option was provided to ensure that participants had an opportunity to report if they did not have an opinion/attitude on a particular statement, rather than feel obliged to provide an answer. The authors acknowledge that while this is an acceptable approach [48], the language used for the ‘don’t know’ response has been debated in the literature.

A possible limitation concerns the development of the framework for integrating deloading into strength and physique sports training programmes (Fig. 2) which has been developed to act as guidance for coaches and sports scientists on the design and implementation of deloading training. While the information located in this framework remained as faithful as possible to the statements presented to the participants, it would be practically meaningful if a fourth round had been included, where participants could provide feedback on the framework to assess its accuracy and usability. Doing so might enhance the clarity of presentation, application and understanding of the framework in practice. Therefore, we recommend that future research should seek coaches’ opinions on the framework presented in Fig. 2 and make necessary revisions to its structure and presentation as required.

## Conclusion

This study acquired expert opinion on the integration of deloading in strength and physique sports. Informed by the findings from the study, consensus was acquired for the development of design principles relating to (1) General Perceptions of Deloading; (2) Potential Applications of Deloading; (3) Designing and Implementing Deloading; and (4), Creating an Inclusive Deloading Training Environment. The novel design principles outlined in this study provide a theoretical and coach-informed method for integrating deloading into strength and physique sports training programmes. In this study, we also propose a new definition of deloading.

Despite the expansion of scientific knowledge exploring deloading, more in-depth research is required. While the framework developed in this research enhances the current understanding of deloading, assisting both strength and physique coaches and sports scientists in the design and implementation of deloading training, it is our recommendation that scientists continue to collaborate with coaches and continue to consolidate deloading knowledge.

### Supplementary Information

**Additional file 1.** Round one short answer questions.

## Data Availability

The data that support the findings of this study are available on request from the corresponding author [LB]. The data are not publicly available due to the inclusion of information that could compromise the privacy of research participants.

## Abbreviations

NFOR: Non-functional overreaching
OTS: Overtraining syndrome
1-RM: One-repetition maximum

## References

[1] Helms ER, Fitschen PJ, Aragon AA, Cronin J, Schoenfeld BJ (2015). Recommendations for natural bodybuilding contest preparation: resistance and cardiovascular training. J Sports Med Phys Fitness.

[2] Suchomel TJ, Nimphius S, Bellon CR, Stone MH (2018). The importance of muscular strength: training considerations. Sports Med.

[3] Turner A (2011). The science and practice of periodization: a brief review. Strength Cond J.

[4] Bell L, Ruddock A, Maden-Wilkinson T, Rogerson D (2020). Overreaching and overtraining in strength sports and resistance training: a scoping review. J Sports Sci.

[5] Grandou C, Wallace L, Impellizzeri FM, Allen NG, Coutts AJ (2020). Overtraining in resistance exercise: an exploratory systematic review and methodological appraisal of the literature. Sports Med.

[6] Meeusen R, Duclos M, Foster C, Fry A, Gleeson M, Nieman DC (2013). Prevention, diagnosis and treatment of the overtraining syndrome: Joint consensus statement of the European College of Sport Science (ECSS) and the American College of Sports Medicine (ACSM). Eur J Sports Exerc Sci.

[7] Mujika I, Padilla S (2000). Detraining: loss of training-induced physiological and performance adaptations. Part I: short term insufficient training stimulus. Sports Med.

[8] Stone MH, Hornsby WG, Haff GG, Fry AC, Suarez DG, Liu J (2021). Periodization and block periodization in sports: emphasis on strength-power training—a provocative and challenging narrative. J Strength Cond Res.

[9] Travis SK, Mujika I, Gentles JA, Stone MH, Bazyler CD (2020). Tapering and peaking maximal strength for powerlifting performance: a review. Sports.

[10] Winwood PW, Dudson MK, Wilson D, Mclaren-Harrison JKH, Redjkins V, Pritchard HJ (2018). Tapering practices of strongman athletes. J Strength Cond Res.

[11] Winwood P, Keogh J, Travis K, Pritchard H (2023). The tapering practices of competitive weightlifters. J Strength Cond Res.

[12] Pritchard HJ, Tod DA, Barnes MJ, Keogh JW, McGuigan MR (2016). Tapering practices of New Zealand’s elite raw powerlifters. J Strength Cond Res.

[13] Grgic J, Mikulic P (2017). Tapering practices of croatian open-class powerlifting champions. J Strength Cond Res.

[14] Escalante G, Stevenson SW, Barakat C, Aragon AA, Schoenfeld BJ (2021). Peak week recommendations for bodybuilders: an evidence based approach. BMC Sports Sci Med Rehabil.

[15] Schoenfeld BJ, Alto A, Grgic J, Tinsley G, Haun CT, Campbell BI (2020). Alterations in body composition, resting metabolic rate, muscular strength, and eating behavior in response to natural bodybuilding competition preparation: a case study. J Strength Cond Res.

[16] Bompa TO, Buzzichelli C (2018). Periodization: theory and methodology of training.

[17] Cunanan AJ, DeWeese BH, Wagle JP, Carroll KM, Sausaman R, Hornsby WG (2018). The general adaptation syndrome: a foundation for the concept of periodization. Sports Med.

[18] Plisk SS, Stone MH (2003). Periodization strategies. Strength Cond J.

[19] DeWeese BH, Hornsby G, Stone M, Stone MH (2015). The training process: planning for strength–power training in track and field. Part 1: theoretical aspects. J Sport Health Sci.

[20] Israetel M, Feather J, Faleiro TV, Juneau CE (2020). Mesocycle progression in hypertrophy: volume versus intensity. Strength Cond J.

[21] Vann CG, Haun CT, Osburn SC, Romero MA, Roberson PA, Mumford PW (2021). Molecular differences in skeletal muscle after 1 week of active versus passive recovery from high-volume resistance training. J Strength Cond Res.

[22] Bell L, Nolan D, Immonen V, Helms E, Dallamore J, Wolf M (2022). “You can’t shoot another bullet until you’ve reloaded the gun”: Coaches’ perceptions, practices and experiences of deloading in strength and physique sports. Front Sports Act Living.

[23] Haugen T (2021). Best-practice coaches: an untapped resource in sport-science research. Int J Sports Physiol Perform.

[24] Rothwell M, Davids K, Stone J, Oullivan M, Vaughan J, Newcombe D (2020). A department of methodology can coordinate transdisciplinary sport science support. J Expert.

[25] Baffour-Awuah B, Pearson MJ, Smart NA, Dieberg G (2022). Safety, efficacy and delivery of isometric resistance training as an adjunct therapy for blood pressure control: a modified Delphi study. Hypertens Res.

[26] Kompf JM, Rhodes RE, Lee S (2022). Selecting resistance training exercises for novices: a Delphi study with expert consensus. Am J Lifestyle Med.

[27] McCall A, Pruna R, Van der Horst N, Dupont G, Buchheit M, Coutts AJ (2020). Exercise-based strategies to prevent muscle injury in male elite footballers: an expert-led delphi survey of 21 practitioners belonging to 18 teams from the big-5 european leagues. Sports Med.

[28] Strafford BW, Davids K, North JS, Stone JA (2022). Feasibility of Parkour-style training in team sport practice: a Delphi study. J Sports Sci.

[29] Villiere A, Mason B, Parmar N, Maguire N, Holmes D, Turner A (2021). The physical characteristics underpinning performance of wheelchair fencing athletes: a Delphi study of Paralympic coaches. J Sports Sci.

[30] Hasson F, Keeney S, McKenna H (2000). Research guidelines for the Delphi survey technique. J Adv Nursing..

[31] Hasson F, Keeney S (2011). Enhancing rigour in the Delphi technique research. Technol Forecast Soc Change..

[32] Holloway K (2012). Doing the E-Delphi: using online survey tools. CIN Comput Inform Nursing..

[33] Bahl JS, Dollman J, Davison K (2016). The development of a subjective assessment framework for individuals presenting for clinical exercise services: a Delphi study. J Sci Med Sport.

[34] Creswell J, Creswell D (2017). Research design: qualitative, quantitative, and mixed methods approaches.

[35] Akins RB, Tolson H, Cole BR (2005). Stability of response characteristics of a Delphi panel: application of bootstrap data expansion. BMC Med Res Methodol.

[36] Okoli C, Pawlowski SD (2004). The Delphi method as a research tool: an example, design considerations and applications. Inform Manag.

[37] Vogel C, Zwolinsky S, Griffiths C, Hobbs M, Henderson E, Wilkins E (2019). A Delphi study to build consensus on the definition and use of big data in obesity research. Int J Obes.

[38] World Medical Association (2013). World Medical Association Declaration of Helsinki: ethical principles for medical research involving human subjects. JAMA.

[39] Iqbal S, Pipon-Young L (2009). The delphi method. Psychologist.

[40] Smith B, Sparkes A, Smith B, Sparkes A (2016). Qualitative interviewing in the sport and exercise sciences. Routledge handbook of qualitative research in sport and exercise.

[41] Fischer JA, Kelly CM, Kitchener BA, Jorm AF (2013). Development of guidelines for adults on how to communicate with adolescents about mental health problems and other sensitive topics: a delphi study. SAGE Open.

[42] Braun V, Clarke V (2006). Using thematic analysis in psychology. Qual Res Psychol.

[43] Braun V, Clarke V (2019). Reflecting on reflexive thematic analysis. Qualit Res Sport Exerc Health.

[44] Robertson S, Zwolinsky S, Pringle A, McKenna J, Daly-Smith A, White A (2013). ‘It is fun, fitness and football really’: a process evaluation of a football-based health intervention for men. Qualit Res Sport Exerc Health.

[45] Dewey J (1986). Experience and education. Educ Forum.

[46] Guba E, Lincoln Y, Denzin N, Lincoln Y (2005). Paradigmatic controversies, contradictions, and emerging confluences. The sage handbook of qualitative research.

[47] Jorm AF (2015). Using the Delphi expert consensus method in mental health research. Aust N Z J Psychiatry.

[48] Lavrakas PJ (2008). Encyclopedia of survey research methods (Vols 1–0).

[49] Runswick OR, Ravensbergen RHJC, Allen PM, Mann DL (2021). Expert opinion on classification for footballers with vision impairment: towards evidence-based minimum impairment criteria. J Sports Sci.

[50] Duffield C (1993). The Delphi technique: a comparison of results obtained using two expert panels. Int J Nurs Stud.

[51] Mujika I, Padilla S (2003). Scientific bases for precompetition tapering strategies. Med Sci Sports Exerc.

[52] Neary JP, Martin TP, Reid DC, Burnham R, Quinney HA (1992). The effects of a reduced exercise duration taper programme on performance and muscle enzymes of endurance cyclists. Eur J Appl Physiol Occup Physiol.

[53] Kreher JB (2016). Diagnosis and prevention of overtraining syndrome: an opinion on education strategies. Open Access J Sports Med.

[54] Bell L, Ruddock A, Maden-Wilkinson T, Hembrough D, Rogerson D (2021). “Is it overtraining or just work ethic?”: Coaches’ perceptions of overtraining in high-performance strength sports. Sports.

[55] Bache-Mathiesen LK, Andersen TE, Dalen-Lorentsen T, Clarsen B, Fagerland MW (2022). Assessing the cumulative effect of long-term training load on the risk of injury in team sports. BMJ Open Sport Exerc Med.

[56] Richardson DB (2009). Latency models for analyses of protracted exposures. Epidemiology.

[57] Soligard T, Schwellnus M, Alonso JM, Bahr R, Clarsen B, Dijkstra HP (2016). How much is too much? (Part 1) International Olympic Committee consensus statement on load in sport and risk of injury. Br J Sports Med.

[58] Gabbett TJ (2016). The training—injury prevention paradox: should athletes be training smarter and harder?. Br J Sports Med.

[59] Eckard TG, Padua DA, Hearn DW, Pexa BS, Frank BS (2018). The relationship between training load and injury in athletes: a systematic review. Sports Med.

[60] Aasa U, Svartholm I, Andersson F, Berglund L (2017). Injuries among weightlifters and powerlifters: a systematic review. Br J Sports Med.

[61] Keogh JWL, Winwood PW (2017). The epidemiology of injuries across the weight-training sports. Sports Med.

[62] Bartolomei S, Hoffman JR, Merni F, Stout JR (2014). A comparison of traditional and block periodized strength training programs in trained athletes. J Strength Cond Res.

[63] Painter KB, Haff GG, Ramsey MW, McBride J, Triplett T, Sands WA (2012). Strength gains: block versus daily undulating periodization weight training among track and field athletes. Int J Sports Physiol Perform.

[64] Pistilli E, Kaminsky DE, Totten L, Miller D (2008). Incorporating one week of planned overreaching into the training program of weightlifters. Strength Cond J.

[65] Redman KJ, Connick MJ, Beckman EM, Kelly VG (2021). Monitoring prescribed and actual resistance training loads in professional rugby league. J Strength Cond Res.

[66] Winwood PW, Cronin JB, Posthumus LR, Finlayson SJ, Gill ND, Keogh JWL (2015). Strongman versus traditional resistance training effects on muscular function and performance. J Strength Cond Res.

[67] Androulakis-Korakakis P, Michalopoulos N, Fisher JP, Keogh J, Loenneke JP, Helms E (2021). The minimum effective training dose required for 1rm strength in powerlifters. Front Sports Act Living..

[68] Iversen VM, Norum M, Schoenfeld BJ, Fimland MS (2021). No time to lift? Designing time-efficient training programs for strength and hypertrophy: a narrative review. Sports Med.

[69] Mujika I, Halson S, Burke LM, Balagué G, Farrow D (2018). An Integrated, multifactorial approach to periodization for optimal performance in individual and team sports. Int J Sports Physiol Perform.

[70] Peake JM, Neubauer O, Della Gatta PA, Nosaka K (2017). Muscle damage and inflammation during recovery from exercise. J Appl Physiol.

[71] Haff GG (2004). Roundtable discussion: periodization of training—part 1. Strength Cond J..

[72] Kiely J (2018). Periodization theory: confronting an inconvenient truth. Sports Med.

[73] Afonso J, Rocha T, Nikolaidis PT, Clemente FM, Rosemann T, Knechtle B (2019). A systematic review of meta-analyses comparing periodized and non-periodized exercise programs: why we should go back to original research. Front Physiol.

[74] Kataoka R, Vasenina E, Loenneke J, Buckner SL (2021). Periodization: variation in the definition and discrepancies in study design. Sports Med.

[75] Evans JW (2019). Periodized resistance training for enhancing skeletal muscle hypertrophy and strength: a mini-review. Front Physiol.

[76] Moesgaard L, Beck MM, Christiansen L, Aagaard P, Lundbye-Jensen J (2022). Effects of periodization on strength and muscle hypertrophy in volume-equated resistance training programs: a systematic review and meta-analysis. Sports Med.

[77] Hackett DA (2022). Training, supplementation, and pharmacological practices of competitive male bodybuilders across training phases. J Strength Cond Res.

[78] Ogasawara R, Yasuda T, Sakamaki M, Ozaki H, Abe T (2011). Effects of periodic and continued resistance training on muscle CSA and strength in previously untrained men. Clin Physiol Funct Imaging.

[79] Ogasawara R, Yasuda T, Ishii N, Abe T (2013). Comparison of muscle hypertrophy following 6-month of continuous and periodic strength training. Eur J Appl Physiol.

[80] Jacko D, Schaaf K, Masur L, Windoffer H, Aussieker T, Schiffer T (2022). Repeated and interrupted resistance exercise induces the desensitization and re-sensitization of mTOR-related signaling in human skeletal muscle fibers. Int J Mol Sci.

[81] Larsen S, Kristiansen E, van den Tillaar R (2021). Effects of subjective and objective autoregulation methods for intensity and volume on enhancing maximal strength during resistance-training interventions: a systematic review. PeerJ.

[82] Greig L, Stephens Hemingway BH, Aspe RR, Cooper K, Comfort P, Swinton PA (2020). Autoregulation in resistance training: addressing the inconsistencies. Sports Med.

[83] Shattock K, Tee JC (2022). Autoregulation in resistance training: a comparison of subjective versus objective methods. J Strength Cond Res.

[84] Helms ER, Storey A, Cross MR, Brown SR, Lenetsky S, Ramsay H (2017). RPE and velocity relationships for the back squat, bench press, and deadlift in powerlifters. J Strength Cond Res.

[85] Thompson SW, Rogerson D, Ruddock A, Barnes A (2020). The effectiveness of two methods of prescribing load on maximal strength development: a systematic review. Sports Med.

[86] Hickmott LM, Chilibeck PD, Shaw KA, Butcher SJ (2022). The effect of load and volume autoregulation on muscular strength and hypertrophy: a systematic review and meta-analysis. Sports Med Open.

[87] Scroggs K, Simonson SR (2021). Writing a needs analysis: exploring the details. Strength Cond J.

[88] Till K, Muir B, Abraham A, Piggott D, Tee J (2019). A framework for decision-making within strength and conditioning coaching. Strength Cond J.

[89] Balagué N, Torrents C, Hristovski R, Kelso JAS (2017). Sport science integration: an evolutionary synthesis. Eur J Sport Sci.

[90] Reade I, Rodgers W, Hall N (2008). Knowledge transfer: How do high performance coaches access the knowledge of sport scientists?. Int J Sports Sci Coach.

[91] Walker LF, Thomas R, Driska AP (2018). Informal and nonformal learning for sport coaches: a systematic review. Int J Sports Sci Coach.

[92] Shaw MP, McNamara SWT (2021). “I can Just Get all the Bits That I Need”: practitioners’ use of open-access sport science podcasts. Front Educ.

[93] Drew C (2017). Educational podcasts: a genre analysis. E-Learn Digit Media.

[94] McNamara S, Larocca V, Bassett-Gunter R (2022). Physical education podcasts: a thriving community of practice or a one-way mode of communication?. Phys Educ Sport Pedagogy..

[95] Waters A, Phillips E, Panchuk D, Dawson A (2019). The coach–scientist relationship in high-performance sport: biomechanics and sprint coaches. Int J Sports Sci Coach.

[96] Chow JY (2013). Nonlinear learning underpinning pedagogy: evidence, challenges, and implications. Quest.

[97] Vernon W (2009). The Delphi technique: a review. Int J Therapy Rehabil.

