# Supplementary material for: Integrating Deloading into Strength and Physique Sports Training Programmes: An International Delphi Consensus Approach
Source: Sports Med Open. 2023 Sep 21;9:87. doi: 10.1186/s40798-023-00633-0 (PMC10511399; doi:10.1186/s40798-023-00633-0)
Supplement: Supplementary file 1 — Additional file 1. Round one short answer questions. [file 40798_2023_633_MOESM1_ESM.docx]

**ROUND ONE SHORT ANSWER QUESTIONS**

**General Perceptions of Deloading**

In your own words, and based on any experiences you may have, please describe what you think ‘deloading’ is?

Have you previously accessed any educational resources about deloading? If you have, what resources did you access?

**Potential Applications of Deloading**

Do you think there is any value in deloading for athlete development? Please expand on your reasons for why or why not.

Do you think there is any value in incorporating deloading into an athlete's training programme when preparing athletes for competitive performance? Please expand on your reasons for why or why not.

**Designing and Implementing Deloading Training**

Do you use deloading into your own coaching practice? Please expand on your reasons for why or why not.

What part of a training programme would deloading be integrated? Please expand on your reasons for why or why not.

How would you adapt the athlete's training volume during deloading? Please expand on your reasons for why or why not, and give specific examples where appropriate.

How would you adapt the athlete's training intensity/effort during deloading? Please expand on your reasons for why or why not, and give specific examples where appropriate.

How would you adapt the athlete's training frequency during deloading? Please expand on your reasons for why or why not, and give specific examples where appropriate.

How would you adapt the athlete's training session duration during deloading? Please expand on your reasons for why or why not, and give specific examples where appropriate.

How would you adapt the athlete's exercise selection during deloading? Please expand on your reasons for why or why not, and give specific examples where appropriate.

How often would you incorporate a deload into the athlete's training programme? Please expand on your reasons for why or why not, and give specific examples where appropriate.

Do you preplan an athlete's deload or do you use an autoregulatory approach? Please expand on your answer, and give specific examples where appropriate.

Do you programme deloading any differently between your own training and your athlete's training?

**Creating an Inclusive Deloading Learning Environment**

Do you think deloading training is easy or difficult to integrate into the sport(s) you are currently working in? Please expand on why you think it is easy or difficult to integrate.

In your opinion, are there any barriers to integrating deloading into your sport? Please expand on your answer in as much detail as possible.

Where barriers exist, do you think these can be overcome? If these barriers can be overcome, how might this be achieved? If these potential barriers cannot be overcome, why not?

**ROUND TWO AND THREE SHORT STATEMENTS**

**DIMENSION: General Perceptions of Deloading**

HIGER ORDER THEME: Deloading Training Typology:

1. Deloading could be a reduction in overall training demand.
2. Deloading could be a reduction in training intensity.
3. Deloading could be a reduction in training volume.
4. Deloading could be a reduction in training frequency.
5. Deloading could be a reduction in proximity to failure.
6. Deloading could be a way of mitigating physical fatigue in the training cycle.
7. Deloading could be a way of mitigating psychological fatigue in the training cycle.
8. Deloading could be a period of recovery.
9. Deloading could be a period of adaptation.

HIGHER ORDER THEME: Taper or Deload?:

1. Deloading is a form of tapering.
2. Deloading is different from tapering, as tapering decreases training volume but maintains training intensity whereas deloading does not.
3. Tapering occurs before competition whereas deloading can occur anywhere in the training programme.
4. Tapering is designed to achieve peaking whereas deloading is designed to promote recovery.

HIGHER ORDER THEME: Current Education on Deloading Training:

1. Deloading is sufficiently represented in strength and conditioning textbooks.
2. Deloading is sufficiently represented in strength and conditioning qualifications.
3. Current approaches to deloading are primarily based on coach experiential knowledge of deloading.
4. There are limited scientific studies and understanding about deloading.
5. Scientific studies on deloading would inform my approach to deloading.
6. Scientific studies on deloading should be open-access.
7. Networking would provide opportunities for coaches to learn more about deloading.
8. Non-traditional media, like blogs, podcasts and YouTube videos could be used to disseminate knowledge of deloading.

**DIMENSION: Potential Applications of Deloading Training**

HIGHER ORDER THEME: Potential Reasons to Deload an Athlete

1. Deloading could increase adherence to normal training.
2. Deloading around competition could allow athletes performance to peak.
3. Deloading could be used to reduce injuries.
4. Deloading could be used to reduce the risk of overreaching.
5. Deloading could be used to reduce the risk of overtraining.
6. Deloading could be used to reduce monotony of training.

**DIMENSION: Designing and Implementing Deloading Training**

HIGHER ORDER THEME: Approaches to integrating deloading into an athlete’s training.

1. Deloading could be coach-driven.
2. Deloading could be athlete-driven.
3. Deloading could be data-driven.
4. Deloading could be a natural break in training.
5. Deloading could be used so intensity of training can be increased afterwards.
6. Deloading could be used so new training blocks and exercises can be introduced.
7. Deloading could occur in the first week of a new mesocycle.
8. Deloading could occur in the last week of a mesocycle.
9. Deloading could occur at the beginning, middle and end of the mesocycle.
10. Deloading could be introduced between mesocycles of training.
11. The timing of deloading depends on competition.
12. Deloading could be planned into the normal training cycle when preparing athletes for competition.
13. Deloading could only be used if training throughout the entire macrocycle is sufficient to drive adaptations.
14. Deloading could occur when athletes feel physically and mentally fatigued regardless of training week in the mesocycle.
15. All parts of the training programme could be deloads.
16. Deloading could be difficult to implement due to sport demands.

HIGHER ORDER THEME: Deload training volume

1. Deloading could be used to maintain fitness.
2. Deloading could reduce training volume by cutting days training.
3. Deloading could reduce volume by lowering sets.
4. Deloading could reduce volume by lowering reps.
5. Deloading could reduce volume from maintenance level.
6. Deloading could use a minimum effective does for volume.

HIGHER ORDER THEME: Deload training intensity and effort

1. Training intensity could remain high during the deload.
2. Training intensity during the deload may not be the same as normal training.
3. Deloading could reduce sets to lower training intensity.
4. Deloading could reduce reps to lower training intensity.
5. Fatigue is related to training intensity.
6. Training intensity during the deload may depend on the demand of the sport.
7. Training intensity during the deload may depend on if the athlete is male or female.
8. Training intensity during the deload may depend on the age of the athlete.
9. Training intensity during the deload may be set using basic exercises.
10. Training intensity could remain the same during the deload but the volume may drop.
11. Deload training intensity could be lower during the first week of a new mesocycle.
12. Deload training intensity could drop over consecutive days.
13. Deload training intensity could remain the same as during normal training.
14. During competitive periods volume and training intensity during the deload may be high to allow recovery.
15. During non-competitive periods volume and training intensity during the deload could decrease.

HIGHER ORDER THEME: Deload training exercise selection

1. During deloading exercise selection could remain unchanged.
2. During deloading the exercise selection may be the athlete decision.
3. During deloading new exercises could be introduced.
4. During deloading, exercise complexity could be introduced.
5. During deloading, ‘pivot blocks’ could be introduced.
6. During deloading, main exercises could be trained with lower volume or training intensity.
7. Deloading could use the same exercises to avoid muscle soreness caused by novel stimulus.
8. Deloading could introduce new exercises that will be used in the next mesocycle.
9. Assistance exercises could be removed during deloading.
10. Training accessories could be removed during deloading.
11. Exercise selection during deloading could match the upcoming mesocycle.
12. Deloading could be used (re)establish technique.
13. Deloading could focus on removing/reducing secondary exercises.
14. Deloading could integrate accessory movements.
15. Deloading reduce volume by decreasing the number of exercises in a training session.
16. Deloading reduces volume by omitting power exercises.
17. Deloading could maintain training demand.
18. Deloading could use familiar exercises.
19. Deloading could use new exercises.
20. Deloading could focus on technique of main lifts.
21. Deloading could keep multijoint exercises.
22. Deloading could include activities outside of the gym .

HIGHER ORDER THEME: Frequency of deloading training

1. Deloading could be included in each mesocycle.
2. There may be multiple deloads depending on the mesocycle length.
3. Deloading frequency may depend on other sports training.
4. If the sport has predominately technical training a deload may not be needed.
5. Deload frequency may depend on how athlete responses to training stimulus.
6. Deloading may occur before, during or at the end of a competition period.

HIGHER ORDER THEME: Autoregulatory and Pre-planned deload training

1. Deloading could be pre-planned.
2. Deloading could be pre-planned around lifestyle.
3. Deloading could be autoregulatory.
4. Deloading could be both pre-planned and autoregulatory.
5. The coach may schedule periods where deloads could be taken.
6. The athlete may schedule periods where deloads could be taken.

HIGHER ORDER THEME: Adapting normal training frequency during deload training.

1. During deloading normal training frequency may not change unless the athlete’s lifestyle is very busy.
2. During deloading training frequency may not alter.
3. Deloading could reduce the mesocycle duration.

HIGHER ORDER THEME: Adapting session duration during deload training

1. During deloading, session duration could increase.
2. During deloading, session duration could decrease.
3. During deloading, session duration could remain the same.
4. During deloading, sessions may be shorter due to lower volume and intensity.
5. During deloading, training volume may be naturally reduced.

HIGHER ORDER THEME: Deloading as a coach and athlete

1. Deloading may be pre-planned for athletes/clients but more autoregulatory in my own training.
2. Deloading may be more autoregulatory for athletes/clients but pre-planned but in my own training.
3. Deloading may be the same for athletes/clients as my own training.
4. Deloading could more cautious when prescribing to athletes compared to my own training.
5. For deloading, volume and intensity may change depending on experience, age, and level of performance.

**DIMENSION: Creating an Inclusive Deloading Training Environment**

HIGHER ORDER THEME: Potential Facilitators towards integrating deloading training and recommendations for resolution.

1. Deloading may be easier to implement when the sport has infrequent competitions.
2. Deloading may be easier to implement when it can be integrated around sport competitions.
3. Deloading may be harder to implement when the sport has frequent competitions.
4. Deloading may be harder to implement as athletes find it boring.
5. Deloads may be hard to implement as athletes love to train.
6. Deloading could allow coaches to identify the needs analysis of new athletes.
7. Deloading may be harder to implement due to athlete perspectives on what a deload is, versus what it actually includes.
8. Deloading may be harder to implement due to the coach’s perspectives on what a deload is, versus what it actually includes.
9. Members of a coaching team not working in collaborative way could be a barrier to integrating deloading.
10. Lack of athlete education and understanding on a deloading could be barrier to integration.
11. Lack of coach education and understanding on a deloading could barrier to integration.
12. Age may be a barrier to deloading integration.
13. Training culture may be a barrier to deloading integration.
14. Illness could be a barrier to deloading integration.
15. Athlete lifestyle could be a barrier to deloading integration.
16. Deloading should be different for males and females.
17. Developing coach education could resolve barriers for integrating deloading.
18. Developing athlete education could resolve barriers for integrating deloading.
19. Working a multi-disciplinary team could reduce barriers for integrating deloading.
20. Involving athletes in decision making on deloading could aid its integration.
21. Research informed practice could aid the integration of deloading.
22. Communication between parties may be key to integrating deloading.
23. Athlete autonomy may be key for integrating deloading.
24. Coaches could focus on relaying the concepts and foundations of deloading to athletes.
25. Coaches could focus on encouraging deloading as a training tool rather than a ‘fad’.
26. Deloading could be integrated in moderation based on the athlete/client training goal.
27. Using consistent terminology when disseminating information on deloading could aid its integration.
